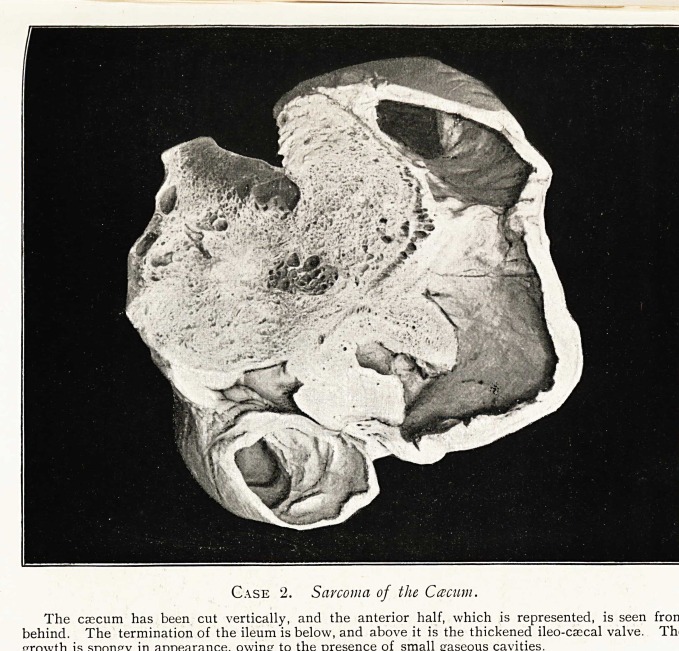# Two Cases of Sarcoma of the Intestine, with Secondary Infection in One by a Gas-Forming Bacillus

**Published:** 1901-03

**Authors:** Theodore Fisher

**Affiliations:** Pathologist to the Bristol Royal Infirmary; Physician to Out-Patients to the Bristol Royal Hospital for Sick Children and Women.


### TWO CASES OF SARCOMA OF THE INTESTINE, WITH SECONDARY INFECTION IN ONE BY A GAS-FORMING BACILLUS.

BY

Theodore Fisher, M.D., M.R.C.P.,

*Pathologist to the Bristol Royal Infirmary; Physician to Out-Patients to the Bristol Royal Hospital for Sick Children and Women.*

---

Sarcoma of the intestines is of sufficient rarity to justify the publication of any case that may occur, and one of the following cases possesses features of some interest not directly dependent upon the presence of the growth.

Two cases of this disease occurred in the Bristol Royal Infirmary in the year 1898. The following are abstracts from the notes:—

A boy, aged 5 years, was admitted, under the care of Dr. Prowse, May 14th, 1898. About three weeks before admission swelling of the abdomen had been noticed by his mother. On admission the boy was rather thin. The abdomen was very distended, and mainly occupying the right half a hard mass with irregular surface could be felt. No signs of free peritoneal fluid were present. There was no tenderness and no complaint of abdominal pain. The motions were loose and rather offensive. The physical signs of the lungs and heart presented nothing noteworthy. On May 25th the notes state that the boy had wasted considerably since his admission. He had lost one and a half pounds in weight. The abdomen, however, had much increased in size, and measured two inches more in girth. The diarrhœa had continued. The bowels had been opened five or six times a day. The temperature, which was 99·6° on admission, had generally oscillated between that point and the normal. On May 27th abdominal section was performed, and the abdominal mass found to be a growth. He died the following day. At the autopsy the boy was seen to be very emaciated, with greatly distended abdomen. No superficial lymphatic glands were enlarged. On opening the abdomen, a large mass of growth the size of a child's head was exposed, which was found to involve the lower portion of the ileum, about twenty inches of its length immediately above the ileo-cæcal valve being affected. The growth had caused enormous enlargement of the affected coils, which approached in size the large intestine of an adult. Section into the mass shewed that the growth had caused no obstruction of the lumen; on the contrary, the lumen was for the most part increased in diameter. Where the growth involved one side of the intestine, it projected into the interior, but at no spot sufficiently to obstruct the lumen; on the other hand, where the growth extensively infiltrated the whole circumference of the intestine, the lumen was greatly widened and measured nearly two inches across. At the margin of the growth, invasion of both the submucous and subperitoneal coats occurred in about equal degree. Deep ulceration was present over a small area the size of a two-shilling piece, which had nearly perforated into the peritoneal cavity. Elsewhere the surface of the growth was free from ulceration. The lymphatic glands of the mesentery were only slightly enlarged by secondary growth. The surface of the liver was covered with small white subperitoneal growths, but the interior of the liver was unaffected. Small growths, however, varying from the size of a pin's head to a small marble, were scattered through both kidneys. An interesting feature was extensive infiltration with growth of the subperitoneal tissues of the abdominal wall in contact with the tumour. The growth extended into the pelvis; but the bladder and rectum remained unaffected. The diaphragm immediately to the right of the falciform ligament was extensively infiltrated, and measured at one spot three-eighths of an inch in thickness. Growth extended upwards from this point to the upper part of the anterior mediastinum, the lymphatic glands of which were extensively involved. The pleuræ and lungs were unaffected, but the bronchial glands were enlarged by secondary growths. The right half of the anterior surface of the fibrous pericardium was infiltrated, and several small nodules were scattered beneath the serous pericardium covering the anterior surface of the right ventricle. Microscopical sections showed the growth to be a lympho-sarcoma with scanty reticulum.

In the following case the growth was in the cæcum:—

A man aged 28, an engine-driver, was admitted into the Bristol Royal Infirmary, under the care of Dr. James Swain, November 14th, 1898. Three weeks previously he had noticed a mass in the right iliac region the size of a duck's egg. Severe abdominal pain followed a week later. Shortly after swelling of the gums occurred, from which slight bleeding took place. On admission the patient, a powerful man, was distinctly ill. The skin was of yellowish tint, and scattered over the neck, arms, and

Case 1. *Sarcoma of the Ileum.*

The greater part of the specimen shows the intestine infiltrated with growth. The small portion of unaffected ileum shows a smaller lumen than that of the coil invaded by growth. At one point the growth appears to be obstructing the lumen, but such was not really the case; the intestinal wall there has not been sufficiently infiltrated on one side to prevent it collapsing.

Case 2. *Sarcoma of the Cæcum.*

The cæcum has been cut vertically, and the anterior half, which is represented, is seen from behind. The termination of the ileum is below, and above it is the thickened ileo-cæcal valve. The growth is spongy in appearance, owing to the presence of small gaseous cavities.

front of the chest and abdomen was a fine purpuric rash. A hard, rounded, immovable mass could be felt in the right iliac region, extending as high as the iliac crest and inwards to within an inch of the middle line. The bowels had not been constipated, and during the few days he was in the Infirmary they were opened once or twice a day. Examination of the blood showed no leucocytosis or poikilocytosis. The hæmoglobin was 50 per cent. On November 16th bleeding from the nose occurred, which was only partially arrested by plugging. The temperature was 101·6° on the night of admission, 102° the following day, and reached 104·4° on the 17th, the morning of death. It only once descended below 100° Abstract of autopsy: Although the *post-mortem* examination was made in the month of November, and only twelve hours after death, the subcutaneous tissues were much distended with gas. This was very marked in the neck and scrotum. The abdomen was also enormously distended, and much gas escaped when an incision into it was made. The intestines also were much distended. Only a few ounces of peritoneal fluid were present. In the right iliac fossa was a hard mass about the size of two fists, involving the cæcum. It was of creamy white colour, and showed no sign of hemorrhage. The growth, where not surrounded by peritoneum, was easily separated from the underlying structures. A section of the tumour showed that it affected the whole circumference of the cæcum, but was of much greater thickness on one side than on the other, the wall on the inner aspect being ten or twelve times the diameter of the outer wall. The muscular coat could be traced some distance into the tumour, and from its situation it could be seen that on the outer side the growth lay mainly in the subperitoneal tissues, but on the inner side in the submucous coat. The peritoneum covering the mass was smooth, and showed no trace of peritonitis. The growth extended upwards towards the descending colon for about five inches from the ileo-cæcal valve, and ended somewhat abruptly. It infiltrated the ileum also, but in much slighter degree, for rather more than two inches of its length. The ileo-cæcal valve was considerably thickened, but there was no obstruction of the ileo-cæcal orifice. There was no ulceration of the mucous membrane. A curious feature of the section of the growth was the presence of gaseous cysts, for the most part small but varying in size, and scattered so thickly as to give the growth the appearance of a sponge. A few small subperitoneal growths were present in the mesentery, but the mesenteric lymphatic glands were not affected. There were no secondary growths present elsewhere in the body. There were no subserous petechiæ either in the pleuræ or peritoneum, but one small hemorrhage was present under the visceral pericardium. The spleen, which was greatly enlarged and weighed 21 oz., was so full of gaseous cysts that it floated in water. The liver showed patches of small cysts scattered here and there, and the kidneys were slightly affected. No other organs, however, contained cysts which attracted attention, and in the brain they were carefully searched for.

Aërobic cultures were taken by Dr. Symes, which proved sterile. Unfortunately, no anaërobic cultures were taken. Microscopical examination showed the growth to be a lympho-sarcoma, with more abundant reticulum than in the former specimen. Around the cysts the cells were closely packed together, showing that the cavities had been formed by some central distending force. I did not detect any micro-organisms in sections stained with methylene blue; but one rather faintly stained with methyl-violet showed many bacilli, especially amongst the sarcoma cells around the cystic cavities. The bacilli were often arranged in pairs, and around every bacillus was a clear space, which was apparently an unstained capsule.

A paper by Dr. Libman, dealing comprehensively with the subject of sarcoma of the small intestine, has recently appeared.[1] Four cases which have come under his own observation are recorded, and at the end of the paper three other cases are mentioned as having occurred recently in New York—two of them at the hospital with which Dr. Libman is connected. This experience is very exceptional, as the statistics of pathologists quoted by Dr. Libman show. At Berlin during sixteen years no case occurred. Elsewhere, although very uncommon, the disease has not been quite so rare. At Prague, in thirteen thousand and thirty-six autopsies, thirteen cases of sarcoma of the intestine occurred, and in Vienna during twelve years twelve cases were met with. Dr. Libman refers to the probably infectious nature of lympho-sarcomata, and suggests that the disease may be endemic in New York. It may be more than a coincidence that the two cases that occurred at the Bristol Royal Infirmary should have occurred in the same year. It will be convenient here to refer to a feature in the morbid anatomy of these growths, which supports the view that they owe their existence to some infection. Secondary growths almost invariably occur by local extension, and when not continuous with the primary growth, the structure affected has been in contact with it. Thus in the first case recorded above, where the mass of primary growth had been in contact with the brim of the pelvis, the peritoneum had become extensively affected. Higher up, the peritoneum of the abdominal wall, where in contact with the tumour, had become infected, and from there the growth had spread to the diaphragm, mediastinum, and pericardium. The visceral pericardium, where in contact with growth in the parietal pericardium, had also become infected. This feature was well marked in Dr. Libman's cases. He says: "A careful examination of the specimens showed that the growth extended almost entirely by continuity, or by contact." Before leaving this subject, it must be mentioned also that Flexner has described small bodies seen in microscopical sections of a sarcoma of the intestine, which were thought by him to be

[1] *Am. J. M. Sc.*, 1900, cxx. 309.

protozoa; but in light of the observations and experiments of others are as likely to have been cells of some form of yeast[1]

Sarcoma of the intestine may occur at any age, but is rather more common between thirty and fifty than before and after those ages. It is more common in males: of forty-nine cases, thirty-five were in males, fourteen in females. The most frequent variety of growth is lympho-sarcoma; but the tumour may be a small-celled, spindle-celled, or mixed-celled sarcoma, and more rarely a myo-sarcoma or melano-sarcoma. The way in which metastasis occurs has already been referred to. Such metastasis, however, though usually present, may be absent. A somewhat distinctive feature of the growth in the intestine is its tendency to dilate rather than obstruct the lumen; this is mentioned by Wilks and Moxon,[2] and Libman quotes Baltzer and others as having noticed the same feature. At one spot, in our first case, the lumen was much dilated. In passing, it may be mentioned that Dr. Libman's explanation of this dilatation of the intestine does not seem to be altogether satisfactory. He attributes the enlargement of the lumen to weakening of the intestinal wall, resulting from destruction of the muscularis mucosæ by the growth. It may be instructive to consider, in this connection, points of difference between carcinoma and sarcoma of the intestine. A carcinoma causes obstruction in two ways: firstly, arising from the innermost layer of the intestine, the abundant proliferation of the cells of this epithelial structure may, in rapidly-growing examples, give rise to a tumour which projects inwards and obstructs the lumen; and secondly, in more chronic cases contraction of the fibrous stroma of the growth produces a stricture of the intestine. Sarcoma, on the other hand, originating commonly in the submucous layer, tends to grow more laterally than internally or externally. Being devoid also of the contracting qualities of a carcinoma, when once the whole circumference of the intestine has become involved, increase of growth will produce a widening of the ring of affected intestine and increase of the diameter of the lumen.

[1] *Johns Hopkins Hosp. Rep.*, 1894, iii. 153.

[2] *Pathological Anatomy*, 3rd Ed., 1889, p. 435.

There is nothing characteristic in the clinical features of sarcoma of the intestines. Abdominal pain is common, and the abdomen becomes distended, either by the size of the growth or by associated peritoneal effusion or tympanites. According to Baltzer, the duration of the disease is from two weeks to one and three-quarter years. It is worthy of mention that cases have simulated appendicitis, and one of Dr. Libman's cases was admitted with that diagnosis. In the second of the cases reported above, Dr. James Swain, under whose care the case was, has told me that at first the probability of the tumour and temperature being the result of appendicitis was carefully considered. This case presented other features of interest. During life the hemorrhages associated with a high temperature pointed to some secondary infection. A case curiously similar is recorded by Hilton Fagge.[1] A man, aged 25, admitted into Guy's Hospital with a history of a few weeks' illness, had a high temperature, developed a purpuric rash, and later lost a considerable quantity of blood from the stomach. Five days before death swelling of the face and scrotum appeared, which after death proved to be due to gaseous distension of the subcutaneous tissues. Small sarcomatous growths were present in the kidneys, but the primary growth appeared to have been at the junction of the ileum and cæcum; "the last two inches of the ileum, including the ileo-cæcal valve, were greatly thickened, forming a massive tumour." In the situation of the tumour, the presence of a purpuric rash and of subcutaneous emphysema, the case bears a remarkable resemblance to the above-mentioned case. In one rather interesting detail it differs. Apparently the swelling of the face and scrotum, due to gaseous distension of the subcutaneous tissues, commenced some days before death.

Emphysema of this nature appears to be generally due to the bacillus aerogenes capsulatus. Encapsuled bacilli were present in our case, and we shall probably be right in concluding that the bacillus was the cause of the gaseous cysts present in the tumour and elsewhere. Another point that suggests itself is the possibility of this gas-forming bacillus having been

[1] *Guy's Hosp. Rep.*, 1881, xxv. 1.

the cause, not only of the presence of gas, but of the purpuric rash and associated elevation of temperature. A case is recorded by Hamilton and Yates,[1] where a young man, aged 22, was attacked with a purpuric rash eleven days before death. The temperature became raised, and he lost large quantities of blood from his nose, mouth, and throat. At the autopsy the abdominal organs were emphysematous. The bacillus aërogenes capsulatus was found, but the staphylococcus pyogenes aureus was also present. Most observers doubt whether the bacillus aërogenes capsulatus frequently enters the circulation long before death, or, if it should do so, that it possesses much pathogenic power unless mixed with other micro-organisms. The possibility of gas-forming bacilli entering the blood many days before death has been made clear to my mind by a case I am hardly at liberty to mention. The importance of the presence of other micro-organisms is a somewhat different question. In the above case aërobic cultures proved sterile, and one might argue from that circumstance that the bacillus aërogenes capsulatus was the only micro-organism present. It is difficult, however, to lay stress upon a negative observation, and the experience of others would lead us to believe that probably a mixed infection was present.

In conclusion, it should be stated that other cases have been recorded by Fagge, and by Martin and Hamilton[2], which show that purpura may be associated with sarcomatous growths. The new growth may in some measure be directly responsible for the hemorrhage, but it seems more probable that it acts indirectly, by producing a way of entrance for infection, and also by lowering the resistance of the body to that infection and its consequences.

[1] *Montreal M. J.*, 1897, xxvi. 117.

[2] *J. Exper. M.*, 1896, i. 595.

## Figures and Tables

**Case 1. f1:**
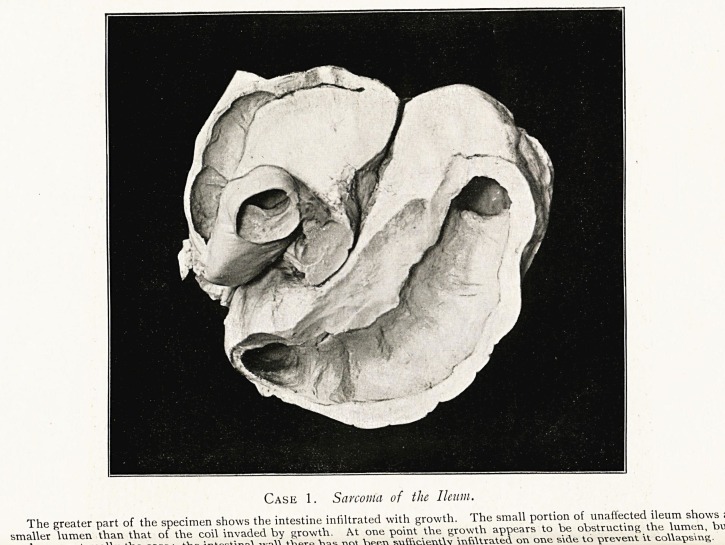


**Case 2. f2:**